# Recent advances in Li_2_S@C nanocomposites for lithium–sulfur batteries

**DOI:** 10.1039/d6sc02457b

**Published:** 2026-06-15

**Authors:** Zhe Huang, Yixuan Zhao, Yonglin Wang, Yuning Li

**Affiliations:** a Department of Chemical Engineering and Waterloo Institute for Nanotechnology (WIN), University of Waterloo, 200 University Ave West Waterloo Ontario N2L 3G1 Canada yuning.li@uwaterloo.ca

## Abstract

Lithium–sulfur batteries (LSBs) are considered as promising next-generation energy-storage systems because of their high theoretical energy density, low cost, material abundance, and environmental compatibility. Over the past decade, intensive research has substantially mitigated key sulfur-cathode limitations, including poor electronic/ionic transport, large volume changes, and the polysulfide shuttle, enabling near-commercial performance in selected studies. These advances have been achieved predominantly in elemental sulfur-based LSBs (S-LSBs), but practical deployment remains largely constrained by reliance on lithium-metal anodes. Lithium sulfide (Li_2_S)-based LSBs (Li_2_S-LSBs) offer an attractive alternative because they can eliminate lithium-metal anodes while retaining the same overall sulfur redox chemistry. However, Li_2_S-LSBs face distinct challenges, most notably the moisture sensitivity of Li_2_S and the high first-charge activation overpotential, which often reduces accessible capacity and compromises cycling stability. The central barrier is the preparation of well-defined Li_2_S@C nanocomposites with Li_2_S uniformly embedded within nanoscale porous carbon hosts, a performance-dictating architecture that is readily achieved for S@C *via* melt infiltration but is difficult for Li_2_S because of its high melting point and limited processability. This review summarizes the current state of Li_2_S@C synthesis, critically comparing major physical and chemical routes (*e.g.*, ball milling, carbothermal methods, lithiation of S@C, sulfuration strategies, solution infiltration, and precursor infiltration–decomposition), and evaluates their advantages, limitations, and scalability. Emerging developments in Li_2_S@C nanocomposites for all-solid-state Li_2_S batteries are also discussed, with emphasis on design strategies for addressing sluggish solid-state reaction kinetics. Finally, we outline complementary directions needed to advance Li_2_S-LSBs toward practical implementation, including Li_2_S-compatible binders and additives that couple shuttle suppression with kinetic promotion, lean-electrolyte cell designs, lithium-free full-cell configurations, and opportunities enabled by integrating Li_2_S@C nanocomposites with solid-state electrolytes.

## Abstract 2

This review highlights synthetic strategies for Li_2_S@C nanocomposites toward lithium-metal-free lithium–sulfur batteries with improved Li_2_S@C activation, sulfur utilization, and solid-state compatibility.

## Graphical abstract

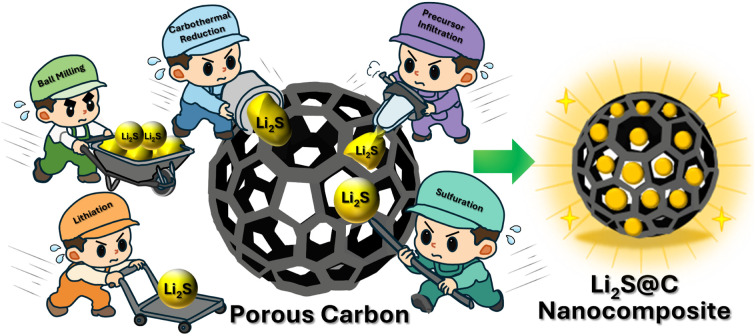

## 1 Introduction

Lithium-ion batteries (LIBs) are widely used in portable electronics, electric vehicles, and grid-level storage.^1–3^ Continued improvements in LIBs based on lithium iron phosphate (LFP), lithium nickel manganese cobalt oxide (NMC), and lithium nickel cobalt aluminum oxide (NCA)^4–7^ have enhanced performance and safety, but further increases in practical cell-level energy density are increasingly constrained by intrinsic material limits and by the cost and supply risks of transition-metal resources.^8,9^ These considerations motivate the exploration of alternative chemistries that can deliver substantially higher energy density with improved sustainability.

Elemental sulfur-based lithium–sulfur batteries (S-LSBs) are leading candidates because sulfur offers an exceptionally high theoretical specific capacity (1672 mA h g^−1^) and is abundant, low-cost, and environmentally benign.^10–13^ When paired with a lithium metal anode, S-LSBs provide a theoretical energy density of 2600 W h kg^−1^ and practical values of 400–600 W h kg^−1^.^11,14,15^ Although the concept of S-LSBs appeared in early patents in the 1960s,^16–18^ which predates that of the Li-intercalation-based rechargeable LIBs first reported in the 1970–1980s,^19,20^ the development of S-LSBs lagged far behind the LIB technology commercialized by Sony in 1991. This gap largely stems from several intrinsic challenges of sulfur cathodes. First, both elemental sulfur (S_8_) and the fully discharged product (Li_2_S) are electronic and ionic insulators, leading to sluggish solid-state redox kinetics. Second, the S_8_ ↔ Li_2_S conversion involves a large volume change (up to ∼78%), which can damage cathode integrity. Third, in commonly used ether-based liquid electrolytes, discharge proceeds through soluble lithium polysulfides (Li_2_S_*x*_, *x* = 4–8). These species can detach from the conductive framework, remain electrochemically inactive in the electrolyte, and migrate to the lithium anode, where they are reduced to insoluble Li_2_S deposits. The resulting loss of active material and parasitic reactions, collectively termed the “polysulfide shuttle”, cause rapid capacity decay and poor coulombic efficiency.

A major advance was reported by Nazar and co-workers in 2009, who mitigated these limitations by thermally infiltrating sulfur into a conductive mesoporous carbon host to form a sulfur-embedded carbon (S@C) nanocomposite.^21^ Nanoscale confinement shortens electron/ion transport pathways, buffers volume changes, and delays polysulfide escape from the cathode. However, because polysulfides interact weakly with nonpolar carbon, shuttling typically persists. Accordingly, extensive efforts have been directed toward strengthening sulfur-species confinement and adsorption using heteroatom-doped carbons,^22–24^ polar/metallic trapping compounds,^25–27^ and functional binders,^28–30^ enabling impressive cycle life (some with ≥80% capacity retention over 1000 cycles). In parallel, covalent immobilization of sulfur in polymeric matrices (*e.g.*, sulfurized polyacrylonitrile, PAN)^31,32^ has proven highly effective for suppressing polysulfide dissolution, while the most definitive strategy is to replace liquid electrolytes with solid electrolytes.^33–35^ Despite these advances, large scale commercialization has not been realized in part because conventional S-LSBs rely on lithium metal anodes, whose high reactivity and dendrite-related safety risks complicate manufacturing and long-term operation.^36–39^

These challenges have stimulated growing interest in Li_2_S-based LSBs (Li_2_S-LSBs), which use Li_2_S, the fully discharged product of sulfur cathodes, as the cathode active material, enabling lithium-metal-free cell configurations.^40–43^ This approach is compatible with existing LIB manufacturing infrastructure and can be paired with high-capacity anode hosts such as silicon and tin. Moreover, Li_2_S undergoes volume shrinkage during delithiation, which can generate internal free volume that partially accommodates subsequent expansion upon lithiation, offering a potentially favorable mechanical pathway for improved cycling stability. Nevertheless, Li_2_S-LSBs introduce new challenges, including the scalable and cost-effective preparation of Li_2_S-embedded carbon (Li_2_S@C) nanocomposites and the high activation overpotential during the first charge, both of which impede practical implementation. While the origin of the first-charge activation overpotential and related mitigation strategies have been reviewed extensively elsewhere,^44^ this article focuses on recent advances in nanostructure engineering of Li_2_S@C nanocomposites, a particularly effective approach for alleviating the activation barrier and addressing other challenges in Li_2_S-LSBs. Through these perspectives, we distill design principles and remaining bottlenecks governing Li_2_S cathode performance and outline research directions toward practically relevant Li_2_S-LSBs.

## 2 Lithium sulfide-based *vs.* sulfur-based Li–S batteries

**Fig. 1:**
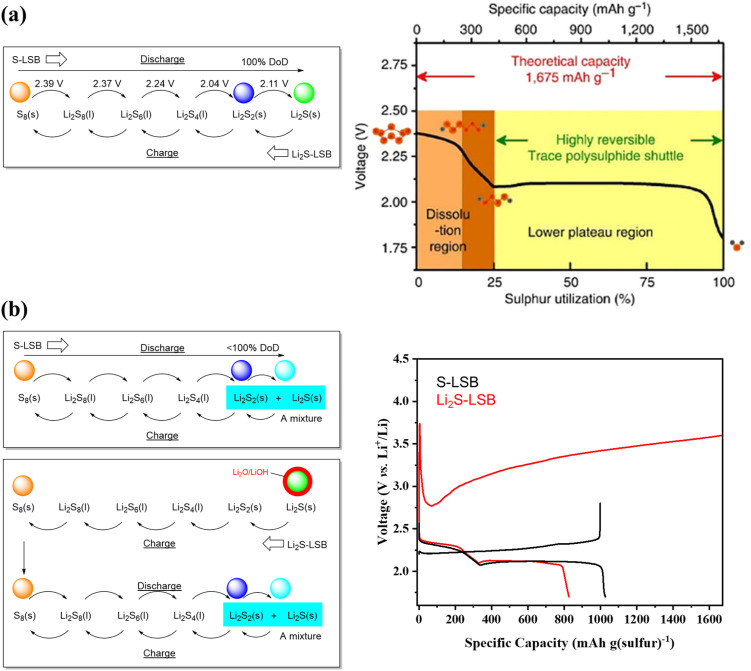
Discharge and charge reactions and galvanostatic discharge curves of (a) ideal S-LSBs and Li_2_S-LSBs,^45^ where DoD is the degree of discharge. Reprinted with permission.^45^ Copyright © 2013, Springer Nature Limited. All rights reserved. (b) Typical real S-LSBs and Li_2_S-LSBs, where specific capacity is based on sulfur (modified from the original graph, showing that, in Li_2_S-LSBs, the first-charge specific capacity often exceeds the theoretical value due to parasitic reactions). Reprinted with permission.^46^ Copyright © 2023 Elsevier B.V. All rights reserved.

In an ether-based liquid electrolyte, the ideal S-LSBs and Li_2_S-LSBs should undergo identical redox cycles and share the same steady-state voltage profile.^44,47–49^ The only difference is the starting point: S-LSBs begin with discharge from elemental sulfur, whereas Li_2_S-LSBs begin with charge from Li_2_S (Fig. 1a). The discharge proceeds through multiple steps and is commonly represented by two plateaus: a shorter high-voltage plateau associated with the conversion of S_8_ to soluble polysulfides (one-quarter of the theoretical capacity) and a longer low-voltage plateau corresponding to the further reduction of polysulfides (*e.g.*, Li_2_S_4_) to insoluble Li_2_S_2_/Li_2_S (three-quarters of the theoretical capacity).

In practice, however, S-LSBs and Li_2_S-LSBs show markedly different first-cycle behaviors (Fig. 1b).^46^ Li_2_S-LSBs using commercial Li_2_S typically require significantly higher charging potentials and exhibit a pronounced initial voltage spike.^44^ This first-charge overpotential is generally attributed to (i) the high thermodynamic stability of crystalline Li_2_S (antifluorite cubic structure), which makes delithiation energetically unfavorable and necessitates the nucleation of sulfur/polysulfide phases;^50–52^ (ii) sluggish interfacial charge transfer and Li^+^ transport through the oxidizing Li_2_S surface region, which further increases the potential at practical rates;^53^ and (iii) passivating surface contaminants on Li_2_S particles (*e.g.*, LiOH/Li_2_O and sometimes Li_2_SO_3_/Li_2_SO_4_) that increase interfacial resistance.^46,54^ By contrast, during the first discharge of an S-LSB, the initial reduction of solid sulfur rapidly generates soluble polysulfides, enabling a favorable solid–liquid pathway and sometimes delivering a near-theoretical first plateau capacity.^25^ The subsequent low-voltage conversion to Li_2_S_2_/Li_2_S is kinetically limited, so the first discharge typically ends with a mixture of poorly crystalline/amorphous Li_2_S_2_/Li_2_S. These freshly formed discharge products also lack the thick impurity layers found on commercial Li_2_S. Therefore, S-LSBs generally delithiate more easily, and the following charge does not exhibit the large activation spike characteristic of Li_2_S-LSBs.

The first-cycle spike of Li_2_S-LSBs can reach ∼4 V *vs.* Li^+^/Li, and the first-charge capacity often significantly exceeds the theoretical value, indicating severe parasitic reactions and/or cathode structural degradation (Fig. 1b). Accordingly, numerous strategies have been developed to mitigate the activation barrier,^44^ including heteroatom/cation–anion doping (*e.g.*, Fe, Co, Se, and Te) to introduce defects, weaken Li–S bonding, and improve transport;^43,55^ incorporation of polar electrocatalysts (*e.g.*, metal sulfides, phosphides, and carbides) to accelerate Li_2_S oxidation;^56–58^ electrolyte additives that remove LiOH/Li_2_O and promote interfacial reactions;^59,60^ and redox mediators that facilitate charge transfer through solution pathways.^61–64^ Reducing Li_2_S crystallinity and particle size can further lower the activation energy and shorten Li^+^ diffusion lengths, improving kinetics and sulfur utilization.^65,66^

As first demonstrated by Nazar and co-workers for S-LSBs,^21^ confining sulfur within a mesoporous conductive carbon host to form an S@C nanocomposite is crucial for achieving high specific capacity and cycle stability by improving electronic/ionic transport, buffering cathode volume changes, and suppressing polysulfide shuttling.^21^ Elemental sulfur can be readily infused into porous carbon by simple melt infiltration (typically at ∼155 °C) above its melting point (∼115 °C). In contrast, Li_2_S has a much higher melting point (∼938 °C), rendering melt infiltration impractical for preparing Li_2_S@C nanocomposites. Moreover, Li_2_S is highly sensitive to moisture, which further complicates synthesis and handling. These challenges have been major obstacles to the development of high-performance Li_2_S-LSBs. In the following sections, representative strategies for preparing Li_2_S@C nanocomposites are introduced and discussed.

## 3 Strategies for preparing Li_2_S@C nanocomposites

Cathodes made from commercial Li_2_S often suffer from high initial charge overpotentials, low capacity, limited rate capability, and poor cycling stability. These issues stem primarily from the large particle size of Li_2_S (10–20 µm),^67,68^ which prolongs Li^+^ diffusion distances, reduces the interfacial reaction area, and aggravates transport and kinetic limitations. In contrast, nanostructured Li_2_S-based cathodes, especially Li_2_S@C nanocomposites, have shown significantly improved electrochemical performance. Here, a Li_2_S@C nanocomposite refers to an architecture in which Li_2_S is confined within, or uniformly distributed throughout, a conductive carbon framework to create intimate and continuous Li_2_S-carbon interfaces. In contrast, Li_2_S/C denotes a simple physical mixture or surface-deposited composite with limited confinement and weaker interfacial integration. The performance improvements of Li_2_S@C nanocomposites arise from nanoscale Li_2_S dispersion in a conductive matrix, which increases the active surface area, shortens ion-transport lengths, and provides efficient electronic pathways for charge transfer.^69^ Equally important, uniform distribution and strong connectivity between Li_2_S and carbon help lower the first-charge activation barrier, accelerate redox kinetics, and increase sulfur utilization.

To realize such structures, a range of synthesis strategies have been developed, including solid-state routes (*e.g.*, high-energy ball milling, carbothermal reduction, and thermal decomposition) and liquid-phase approaches (*e.g.*, precipitation, infiltration, and *in situ* conversion). The following subsections summarize representative preparation strategies, structural designs, and the resulting electrochemical benefits of Li_2_S@C nanocomposites, showing their key role in enabling high-performance and practically relevant Li_2_S-LSBs.

### 3.1 Ball milling

Ball milling is a mechanical technique that utilizes repeated collisions between powder particles and milling media to reduce particle size and induce defects.^70,71^ For Li_2_S cathodes, it has been widely used to downsize commercial Li_2_S and promote intimate mixing with conductive carbon, thereby improving interfacial contact and enhancing electrochemical reactivity.^72^ High-energy ball milling can typically reduce Li_2_S to the submicron range (∼0.2–2 µm), shortening Li^+^ diffusion lengths and facilitating electron transport.

**Fig. 2:**
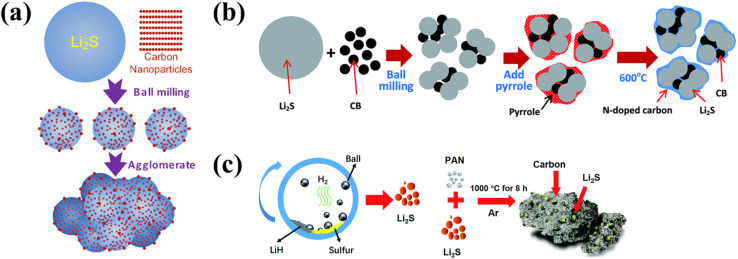
(a) Schematic of the high-energy ball milling process for preparing Li_2_S-C nanocomposites. Reprinted with permission.^73^ Copyright © 2012, American Chemical Society. All rights reserved. (b) Schematic of the approach for synthesizing the Li_2_S/C composite particles encapsulated by a nitrogen-doped carbon shell. Reprinted with permission.^74^ Copyright © 2014, Royal Society of Chemistry. All rights reserved. (c) Schematic illustration of the fabrications of Li_2_S and the Li_2_S/C hybrid. Reprinted with permission.^75^ Copyright © 2017, Royal Society of Chemistry. All rights reserved.

Cai *et al.* prepared a nanostructured Li_2_S/C composite by ball milling commercial Li_2_S with carbon black at 1060 rpm for 2 hours, resulting in particles ranging from 200 to 500 nm as shown in Fig. 2a.^73^ This composite showed a reduced activation potential of 2.6 V (*vs.* Li^+^/Li) compared to commercial Li_2_S; however, high cutoff voltages up to 4.0 V were still required to complete the initial charge. In another study, Chen *et al.* (Fig. 2b) combined high-energy ball milling of Li_2_S and carbon black with pyrrole-assisted carbonization to form ∼400 nm Li_2_S/C particles encapsulated by a N-doped carbon shell.^74^ This core–shell structure delivered a high initial capacity of 1029 mA h g^−1^ and retained 652 mA h g^−1^ after 100 cycles, indicating improved cycling stability.^74^ Similarly, Li *et al.* synthesized nanosized Li_2_S by ball milling a LiH + S_8_ precursor mixture (Fig. 2c).^75^ The Li_2_S particles were subsequently mixed with mesoporous carbon matrices by milling with polyacrylonitrile (PAN), followed by high-temperature carbonization (∼1000 °C), forming conductive and mechanically robust nanocomposites. A practical concern is that the reaction between LiH and S_8_ can generate hydrogen gas (H_2_) during milling, posing a safety risk for scale-up. Ball milling also enables incorporation of functional additives. For example, Cupid *et al.* introduced polar SnS_2_ into Li_2_S/C *via* a scalable milling process to chemically trap lithium polysulfides, suppress shuttling, and stabilize interfacial reactions.^76^

Despite its simplicity, ball milling has notable limitations: it is energy-intensive and time-consuming, achieving uniform Li_2_S dispersions below ∼100 nm remains difficult,^77^ and the process cannot effectively infiltrate Li_2_S into the internal pore network of mesoporous carbon hosts.

### 3.2 Carbothermal reduction

Carbothermal reduction is a high-temperature process in which carbon serves as a reducing agent to convert metal oxides, sulfates, or other precursors into lower-valence products such as metals, sulfides, or carbides under inert or reducing atmospheres (*e.g.*, Ar, N_2_, or H_2_).^78^ In addition to traditional carbon sources such as graphite and carbon black, carbon-rich polymers such as glucose, polyvinyl alcohol (PVA), polyvinylpyrrolidone (PVP), and polyacrylonitrile (PAN) are often used as carbon precursors.^79,80^ Upon thermal decomposition, these polymers generate carbon *in situ*, enabling intimate precursor–carbon contact and thereby enhancing reduction efficiency and compositional control.

In 2013, Yang *et al.* pioneered a cost-effective carbothermal route to synthesize Li_2_S@C composites from Li_2_SO_4_*via* the reaction described in eqn (1):^81^

1 Li_2_SO_4_ (s) + *x*C (s) → Li_2_S (s) + *x*CO_*y*_ (g) ((*x*, *y*) = (1, 1) or (4,2))

**Fig. 3:**
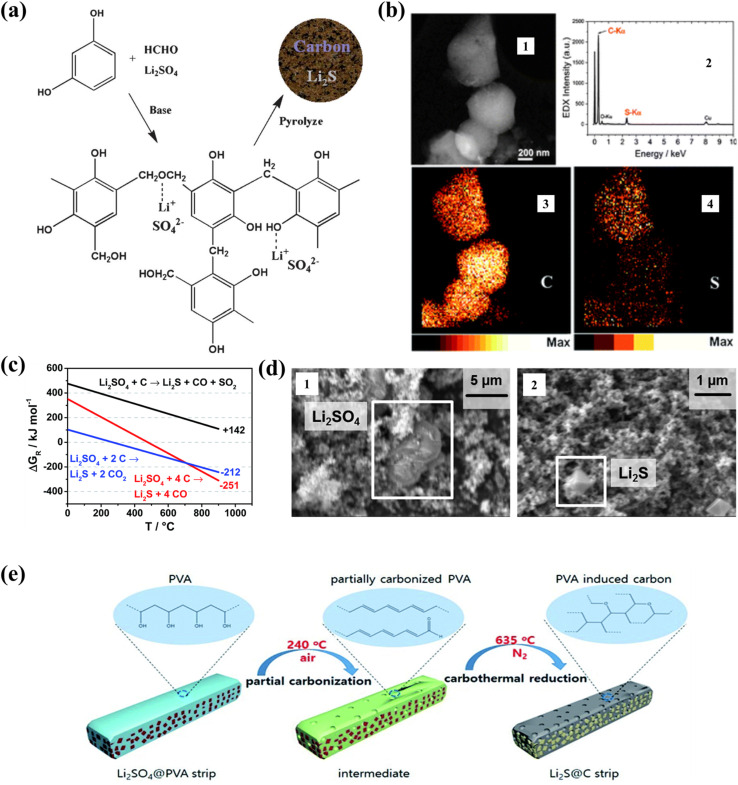
(a) *In situ* synthesis scheme for a Li_2_S@C composite. (b) HAADF-STEM image of Li_2_S@C particles (1), EDX spectrum showing the presence of the carbon K edge and sulfur K edge (2), and EDX elemental mapping of carbon (3) and sulfur (4). Reprinted with permission.^81^ Copyright © 2013, Royal Society of Chemistry. All rights reserved. (c) Ellingham diagram for different carbothermal reduction reactions. The numbers at the end of the lines are Δ*G*_R_ values at 820 °C. (d) SEM images from a Li_2_SO_4_·H_2_O sample before and after heat treatment at 820 °C under an argon atmosphere. Reprinted with permission.^82^ Copyright © 2015, Royal Society of Chemistry. All rights reserved. (e) Schematic of the fabrication process of Li_2_S@C strips and the structural evolution of PVA during PVA-assisted carbothermal reduction of Li_2_SO_4_. Reprinted with permission.^79^ Copyright © 2018, Royal Society of Chemistry. All rights reserved.

As shown in Fig. 3a, the carbon framework is formed by pyrolyzing a resorcinol–formaldehyde (RF) gel, which is synthesized *via* condensation polymerization.^81^ Due to its high surface area, porosity, and conductivity, the abundant oxygen groups in the RF gel can coordinate with Li^+^ in Li_2_SO_4_, promoting uniform distribution of the salt in the carbon matrix. TEM analysis shows that the cross-linked RF-derived carbon forms spherical particles with sizes ranging from ∼500 nm to 2 µm. STEM-EDX mapping in Fig. 3b confirms that sulfur is homogeneously distributed throughout the carbon spheres, indicating successful incorporation rather than surface deposition. The Li_2_S@C composite exhibits higher reversible capacity and significantly improved suppression of the polysulfide shuttle compared to the physical mixture. At 0.5C, it retains 280 mA h g^−1^ after 40 cycles (from 330 mA h g^−1^), demonstrating improved cycling stability, though further optimization is still needed.

Temperature is a key parameter in carbothermal reduction because it strongly influences the crystallinity and particle size of Li_2_S. The Ellingham diagram (Fig. 3c) indicates that reduction of Li_2_SO_4_ by carbon becomes thermodynamically favorable above ∼300 °C.^82^ SEM images (Fig. 3d) reveal a morphological evolution from monoclinic Li_2_SO_4_·H_2_O to well-defined octahedral particles, indicating the successful formation of Li_2_S crystals. Nonetheless, many studies conduct the reaction at around ∼800 °C, which accelerates grain growth and yields larger, highly crystalline particles.^83^ In contrast, Ye *et al.* showed that using PVA as a carbon source and conducting the reaction below the melting point of Li_2_SO_4_ (635 °C) helps preserve morphology and yields smaller Li_2_S particles (10–20 nm), likely due to gradual oxygen removal (Fig. 3e).^79^ The unsaturated C=C and C=O bonds in the partially carbonized polymer enable efficient reduction at considerably low temperatures. Notably, Li_2_S@C prepared under these milder conditions exhibited a reduced activation potential (2.63 V) and a higher initial discharge capacity (805 mA h g^−1^) compared to the material produced at 900 °C (3.2 V and 760 mA h g^−1^).

Carbothermal reduction has several drawbacks. Evolution of CO and CO_2_ gases during reduction can generate excessive porosity in the carbon matrix, decreasing cathode volumetric capacity.^78,82^ The process may also release hazardous sulfur-containing gases (*e.g.*, SO_2_, SO_3_, and H_2_S), raising safety and environmental concerns.^84^ In addition, morphology control of Li_2_S remains challenging, particularly for high-temperature syntheses, where particle coarsening is difficult to suppress.

### 3.3 Carbothermal reduction-derived methods

**Fig. 4:**
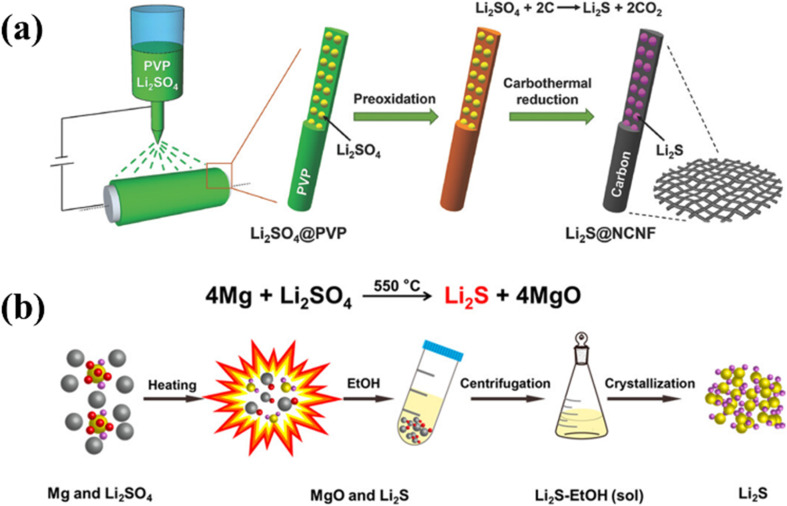
(a) Schematic illustration of the production of freestanding flexible Li_2_S@NCNF paper electrodes *via* Ar-protected carbothermal reduction of Li_2_SO_4_@PVP fabrics made by electrospinning under ambient conditions. Reprinted with permission.^85^ Copyright © 2017 WILEY-VCH Verlag GmbH & Co. KGaA, Weinheim. All rights reserved. (b) Illustrative process of the magnesothermal synthesis of Li_2_S and purification. Reprinted with permission.^89^ Copyright © 2022, American Chemical Society. All rights reserved.

PVP can serve as both a carbon source and structural template in the carbothermal reduction of Li_2_SO_4_ to Li_2_S, forming Li_2_S@C composites. However, conventional solid-state mixing yields inhomogeneous dispersion and poorly controlled morphologies of Li_2_S particles. To address this, electrospinning is used to fabricate uniform Li_2_SO_4_/PVP nanofibers as structured precursors (Fig. 4a).^85^ The nanofiber geometry promotes nanoscale dispersion of Li_2_SO_4_ and creates an interconnected architecture. Subsequent annealing under a reducing atmosphere converts Li_2_SO_4_ into finely distributed Li_2_S within conductive carbon nanofibers, which can be collected as free-standing, binder-free electrodes. A major limitation is the low mass loading of typical electrospun mats, which restricts areal capacity unless multiple layers are stacked. In addition, scaling-up remains challenging due to the limited throughput of traditional electrospinning setups. The solvent system must also be carefully chosen to balance PVP solubility, Li_2_SO_4_ dispersion, and electrospinnability.^86^ Moreover, the final fiber morphology is highly sensitive to processing parameters and ambient conditions, necessitating tight process control.^87,88^

Also inspired by the traditional carbothermal reduction method, the magnesothermal synthesis of Li_2_S replaces carbon with magnesium (Mg) as the reducing agent (Fig. 4b).^89^ While both approaches reduce lithium-sulfur-containing precursors such as Li_2_SO_4_ to Li_2_S, Mg offers a much stronger reduction driving force, allowing the reaction to proceed at lower temperatures and with more favorable thermodynamics. Unlike carbothermal methods, which often generate gaseous byproducts such as CO and CO_2_ and leave highly porous carbon residues, magnesothermal synthesis produces solid MgO as the main byproduct, avoiding greenhouse-gas emissions. After the reaction, high-purity Li_2_S is typically obtained through thorough removal of MgO, and an ethanol-based dissolution/recrystallization step is often employed to improve purity and narrow the particle-size distribution, which helps ensure reproducible electrochemical performance. Despite these advantages, magnesothermal synthesis has practical limitations. Mg powder is pyrophoric and must be handled under inert conditions. The reaction is highly exothermic, complicating heat management and scale-up. After the reaction, MgO byproducts must be fully removed, and an additional ethanol-based recrystallization step is often needed to purify Li_2_S. The method also offers little control over morphology and uniformity.

### 3.4 Lithiation of S@C nanocomposites

**Fig. 5:**
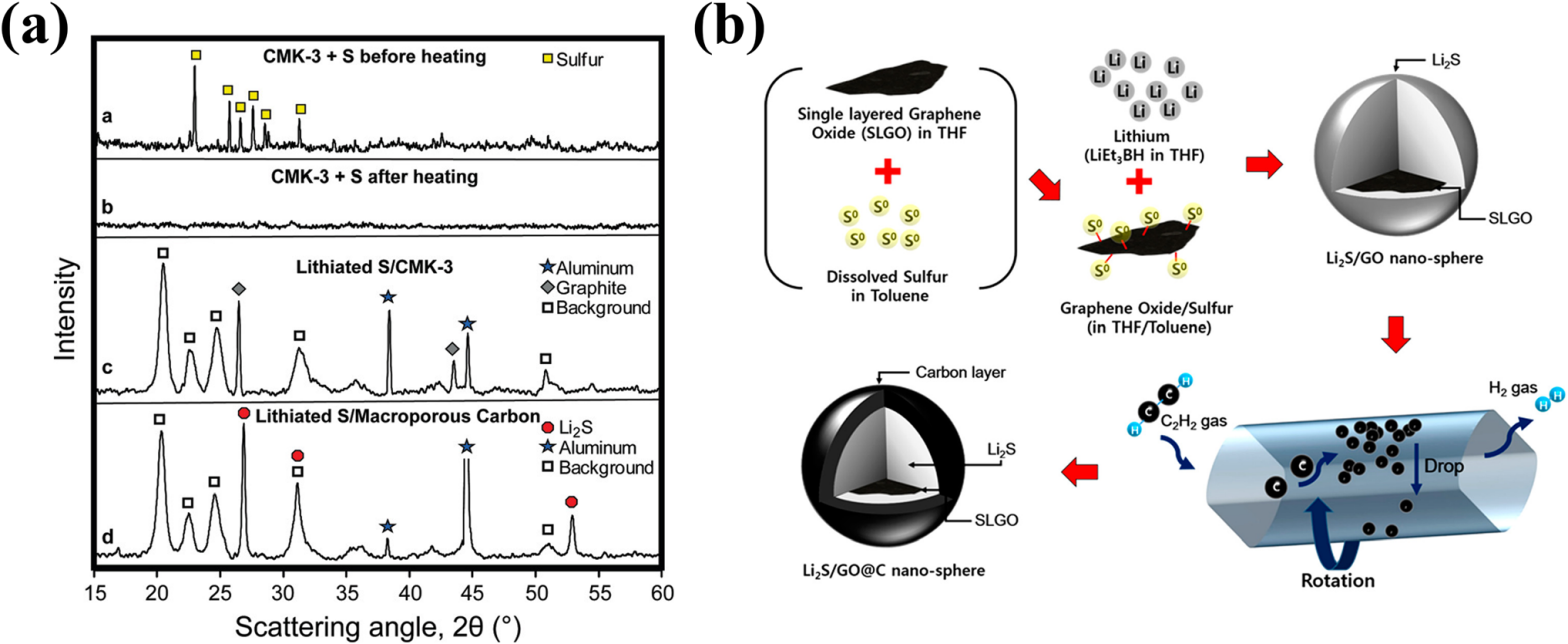
(a) X-ray diffraction characterization of Li_2_S/mesoporous carbon nanocomposite particles. Reprinted with permission.^91^ Copyright © 2010, American Chemical Society. All rights reserved. (b) Schematic illustration of the synthesis of Li_2_S/GO@C nanospheres. Reprinted with permission.^93^ Copyright © 2015, American Chemical Society. All rights reserved.

Li_2_S@C nanocomposites can be directly synthesized by lithiating S@C nanocomposites using lithium metal or reducing lithium reagents (Table 1). For example, Yang *et al.* lithiated a S@CMK-3 nanocomposite prepared by thermal infiltration using *n*-butyllithium at 65 °C for 2 h and then at 105 °C for 18 h (Fig. 5a).^91^ In the XRD pattern of the lithiated product, the diffraction peaks of sulfur disappeared, indicating complete conversion. Notably, no Li_2_S reflections were observed either, which was attributed to Li_2_S being confined within the sub-5 nm CMK-3 pores, thereby suppressing crystallite growth and long-range ordering. To verify Li_2_S formation, the same lithiation was performed on an S/carbon composite based on macroporous carbon (200–300 nm pores). In this case, clear Li_2_S diffraction peaks were observed, supporting Li_2_S formation in S/CMK-3 as well. The composite delivers an initial discharge capacity of 573 mA h g^−1^, with the capacity stabilizing after approximately five cycles. Additionally, a small potential difference of only ∼200 mV between the first charge and subsequent charges further indicates the significantly improved reaction kinetics of Li_2_S upon incorporation into the mesoporous carbon nanocomposite. Hwa *et al.* reported the synthesis of Li_2_S/GO@C nanospheres *via* lithiation of sulfur using LiET_3_BH.^93^ Briefly, an S/single-layer graphene oxide (S/SLGO) nanocomposite was prepared by combining a sulfur solution in toluene with an SLGO dispersion in THF, followed by slow addition into a LiEt_3_BH solution in THF to yield Li_2_S/GO nanospheres after solvent removal. The Li_2_S/GO nanospheres were subsequently treated under hydrogen to generate a carbon shell, producing Li_2_S/GO@C nanospheres (Fig. 5b). The carbon-coated Li_2_S/GO@C and Li_2_S/GO@C-NR electrodes deliver high initial capacities of 964 and 896 mA h g^−1^ (based on Li_2_S), respectively, significantly exceeding those of uncoated electrodes. The carbon shell enhances sulfur utilization by suppressing polysulfide dissolution and improving electronic conductivity.

**Table 1:** Lithiation agents for elemental sulfur and corresponding reactions

Lithiation agent	Formula	Conditions	Reaction	Ref.
Lithium metal	Li	50–70 °C, inert gas, dry ether solvent	S + 2Li → Li_2_S	90
*n*-Butyllithium	C_4_H_9_Li	Room temp., dry THF or hexane, inert atmosphere	S + 2C_4_H_9_Li → Li_2_S + C_4_H_9_ − C_4_H_9_	91
Lithium hydride	LiH	High-energy ball milling	S + 2LiH → Li_2_S + H_2_	75
Lithium naphthalenide	LiC_10_H_8_	In THF, under Ar	S + 2LiC_10_H_8_ →Li_2_S + 2C_10_H_8_	92
Lithium triethylborohydride	LiBEt_3_H	Anhydrous ether solvents, ambient conditions	S + 2LiBEt_3_H → Li_2_S + H_2_ + 2BEt_3_	93 and 94

The chemical lithiation route enables low-temperature synthesis compared to carbothermal approaches and can achieve uniform dispersion of Li_2_S within a conductive carbon matrix, especially when nanostructured carbon hosts are used.^95^ Importantly, this strategy is frequently combined with chemical vapor deposition (CVD) to further engineer the interfacial structure. After sulfur infiltration, or even after partial lithiation, a conformal carbon layer can be deposited *via* CVD to reinforce electronic percolation, seal residual surface defects, and stabilize the newly formed Li_2_S phase.^93,96^ However, the practical adoption of lithiation is constrained by the safety hazards and high cost of typical lithiation reagents, which pose significant barriers to scale-up and commercialization.

### 3.5 Sulfuration of lithium compounds

Sulfuration has emerged as a versatile and potentially scalable route to synthesize Li_2_S from a wide range of lithium-containing precursors. In this approach, reactive sulfur-containing gases, such as H_2_S, CS_2_, and sulfur vapor, convert lithium precursors in the solid, molten, or vapor state into Li_2_S under controlled thermal and atmospheric conditions.

Air-stable lithium salts such as LiOH, Li_2_CO_3_, and LiNO_3_ are attractive starting materials because they are easy to handle and stable under ambient conditions.^97^ In addition, several lithium salts with relatively low melting points, such as lithium nitrite (LiNO_2_, 222 °C) and lithium acetate (CH_3_COOLi, 286 °C),^97^ can be melt-infiltrated into carbon hosts to form Li-salt@C composites, which are subsequently sulfurized to yield Li_2_S@C. Representative conversion reactions are summarized in Table 2.

**Table 2:** Chemical sulfuration agents for lithium-rich precursors

Sulfuration agent	Reaction	Ref.
H_2_S	2ROLi (sol) + H_2_S (g) → 2Li_2_S (s) + H_2_ (g)	97
	2LiX (s) + H_2_S (g) → 2Li_2_S (s) + 2HX (g)	98
CS_2_	4Li (l) + CS_2_ (g) → 2Li_2_S (s) + C (s)	99
	2LiOH (s) + CS_2_ (g) → Li_2_S (s) + CO_2_ (g) + H_2_S (g)	100
	4LiH (s) + CS_2_ (g) → 2Li_2_S (s) + C (s) + 2H_2_ (g)	101
S (gas)	2Li + S (g) → Li_2_S (s)	102
	6LiOH (s) + 3S (g) → 2Li_2_S (s) + Li_2_SO_3_ (g) + 3H_2_O (g)	84

**Fig. 6:**
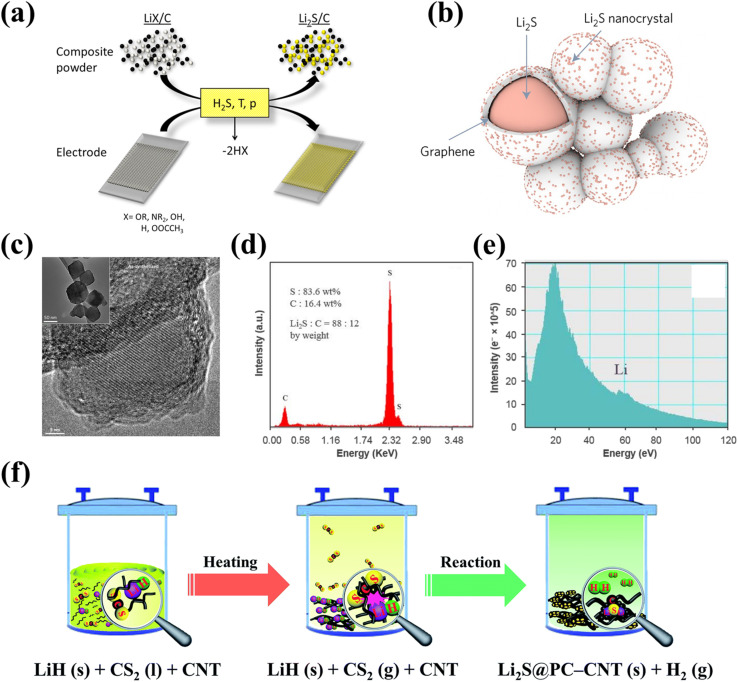
(a) Schematic illustration of routes for preparing Li_2_S/C composite and electrodes. Reprinted with permission.^98^ Copyright © 2016 Elsevier B.V. All rights reserved. (b) Schematic illustration of Li_2_S@graphene capsules by burning lithium in CS_2_. (c) TEM images of Li_2_S@graphene capsules, with the inset showing bulk nanocapsules, revealing single-crystal Li_2_S (50–100 nm) encapsulated by about 10 to 20 graphite layers. (d) EDXS spectra and (e) EELS spectra of Li_2_S@graphene capsules shown in 6c. Reprinted with permission.^99^ Copyright © 2017, Springer Nature Limited. All rights reserved. (f) Schematic illustration of the synthesis procedure for Li_2_S@PC-CNT. Reprinted with permission.^101^ Copyright © 2018, Royal Society of Chemistry. All rights reserved.

The required reaction temperature depends strongly on the lithium precursor: strong Brønsted base salts (*e.g.*, LiOH, LiH, and LiNH_2_) can react at ∼100 °C, while Li_2_CO_3_ and CH_3_COOLi require substantially higher temperatures of ∼400–725 °C. Lower conversion temperatures are generally preferred as they help preserve the original morphology, minimize Li_2_S particle coarsening, and reduce energy consumption. Pre-processing steps such as ball milling or recrystallization can further reduce and homogenize precursor particle size prior to sulfuration. For example, Dressel *et al.* used high-energy ball milling to decrease the particle size of LiOH and then mixed it with carbon black (Fig. 6a).^98^ The LiOH/C mixture was dispersed with a binder to form a slurry, cast onto an aluminum current collector, and subsequently sulfurized under continuous H_2_S gas flow at 100 or 150 °C. The resulting Li_2_S/C electrodes delivered discharge capacities of up to 770 mA h g^−1^ and retained >410 mA h g^−1^ after 100 cycles at 0.2C.

Tan *et al.* synthesized 50–80 nm Li_2_S nanocrystals encapsulated by few-layer graphene (Li_2_S@graphene) *via* combustion of lithium foil in CS_2_ vapor (Fig. 6b).^99^ TEM images reveal rhombic Li_2_S@graphene nanoparticles (50–80 nm) with highly crystalline Li_2_S cores tightly encapsulated by 10–20 layers of graphene, forming a capsule-like core–shell nanostructure (Fig. 6c). The EDXS results confirm such a high active mass percentage, where the Li_2_S/C ratio is determined to be 88 : 12 by weight (Fig. 6d). The EELS results further confirm the presence of the Li element in the Li_2_S@graphene composite (Fig. 6e). At a high Li_2_S loading of 10 mg cm^−2^, the electrode delivers a high reversible capacity of 1160 mA h g^−1^. Liang *et al.* used CS_2_ to sulfurize LiH to Li_2_S at temperatures below 250 °C (Fig. 6f).^101^ As a result, the electrode achieved 820 mA h g^−1^ at 0.1 A g^−1^ after 10 cycles and showed excellent cycling stability, retaining 502 mA h g^−1^ at 0.5 A g^−1^ after 300 cycles (relative to an initial capacity of ∼650 mA h g^−1^ at 0.5 A g^−1^).

The most direct route to Li_2_S is the reaction between lithium and sulfur. Although rarely used in practice due to difficult handling, sulfur vapor can react with Li metal in a sealed vacuum vessel at ∼300 °C to form Li_2_S.^103^

Overall, sulfuration offers several advantages, including the use of air-stable lithium precursors, tunable reaction conditions, and control over Li_2_S particle size and crystallinity. It also facilitates integration with conductive carbon frameworks and is, in principle, scalable. However, the use of toxic and flammable gases such as H_2_S poses significant safety and environmental risks, necessitating specialized reactors and stringent gas-handling infrastructure. For some precursors, high processing temperatures are required, which can promote particle agglomeration and coarsening, compromising electrochemical performance. In addition, incomplete conversion and residual precursors/byproducts often necessitate post-treatment and purification.

### 3.6 Solution infiltration of Li_2_S

**Fig. 7:**
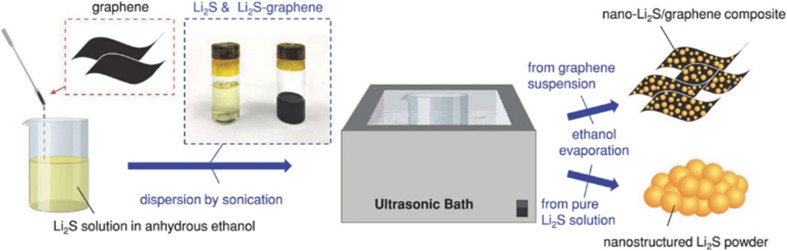
Schematic of the Li_2_S and Li_2_S–graphene composite synthesis process. Reprinted with permission.^104^ Copyright © 2014 WILEY-VCH Verlag GmbH & Co. KGaA, Weinheim. All rights reserved.

**Fig. 8:**
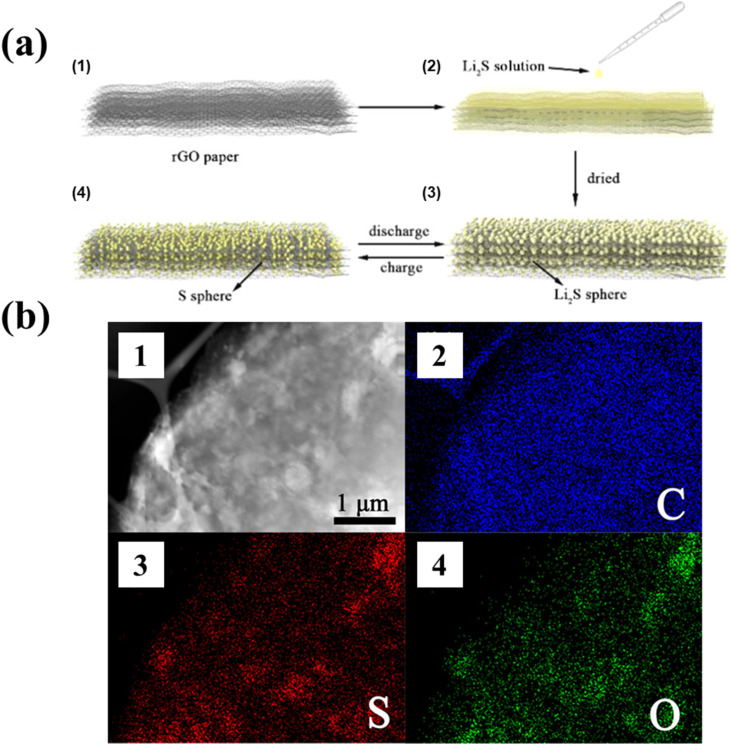
(a) Schematic illustration of the material preparation processes of nano-Li_2_S/rGO paper and structure changes during cycling of nano-Li_2_S/rGO paper. (b) HAADF-STEM image of nano-Li_2_S/rGO paper (1) and corresponding EDS elemental mapping of (2) C, (3) S, and (4) O Reprinted with permission.^105^ Copyright © 2015, American Chemical Society. All rights reserved.

Li_2_S can be dissolved in a suitable solvent and infiltrated into porous carbon hosts to form Li_2_S@C nanocomposites after solvent evaporation. Graphene has been frequently used as the carbon host. In 2014, Wu *et al.* reported a simple solution-based route to graphene–Li_2_S composites.^104^ Briefly, commercial Li_2_S powder was dissolved in anhydrous ethanol, followed by the addition of graphene. Solvent evaporation under continuous mixing deposited Li_2_S onto the graphene sheets, yielding a well-integrated Li_2_S composite (Fig. 7). The Li_2_S–graphene composites exhibited excellent electrochemical performance in both 5 M and 7 M electrolytes. With increasing graphene content and reduced Li_2_S particle size, capacity utilization improved significantly, delivering a maximum specific capacity exceeding 1100 mA h g^−1^ (S) at C/20, comparable to or higher than those of previously reported S–CNT and S–graphene cathodes. Wang *et al.* drop-cast a Li_2_S–ethanol solution onto reduced graphene oxide (rGO) paper to uniformly infiltrate Li_2_S into rGO's porous framework (Fig. 8a).^105^ HAADF-STEM and elemental mapping shown in Fig. 8b confirm the uniform distribution of Li_2_S nanoparticles on graphene sheets, with C, S, and O elements homogeneously dispersed throughout the nano-Li_2_S/rGO structure. The flexible rGO paper not only prevented particle agglomeration but also helped accommodate volume changes during cycling, thereby enhancing structural stability and electrochemical performance.

**Fig. 9:**
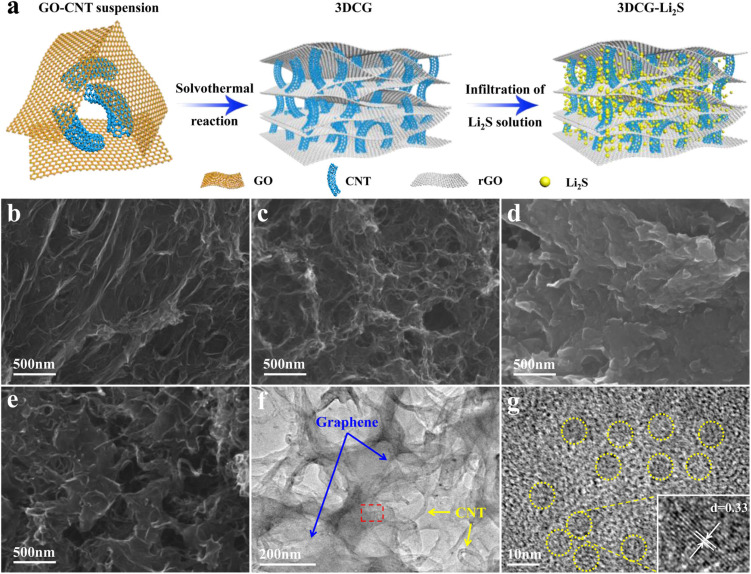
(a) Synthetic procedure for the 3DCG–Li_2_S composite. Reprinted with permission.^106^ Copyright © 2016, American Chemical Society. All rights reserved. SEM images of (b) 3DG, (c) 3DCG, (d) 3DG–Li_2_S, and (e) 3DCG–Li_2_S composite. (f) Low-magnification TEM images of 3DCG–Li_2_S. (g) TEM image of Li_2_S nanoparticles on 3DCG and a high-resolution TEM image of Li_2_S nanocrystals in the inset. Reprinted with permission.^106^ Copyright © 2016, American Chemical Society. All rights reserved.

He *et al.* incorporated carbon nanotubes (CNTs) into a graphene oxide (GO) suspension to construct a three-dimensional conductive network.^106^ As shown in Fig. 9a, the one-dimensional CNTs served as nanoscale pillars that separated and supported the two-dimensional graphene sheets, yielding a robust 3D architecture with enhanced conductivity and mechanical integrity. Li_2_S was then incorporated by infiltrating a Li_2_S–ethanol solution followed by vacuum-assisted evaporation, enabling uniform Li_2_S recrystallization within the interlayer voids. TEM images reveal uniformly dispersed ultrafine Li_2_S nanoparticles (∼8 nm) anchored on the three-dimensional CNT/graphene conductive network, with a lattice spacing of 0.33 nm corresponding to the Li_2_S (111) plane, as shown in Fig. 9b–g.

**Fig. 10:**
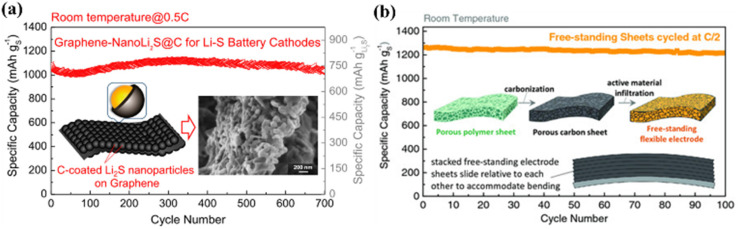
(a) Cycling stability of the graphene–nano Li_2_S@C electrode at 0.5C and its morphological characterization. Reprinted with permission.^107^ Copyright © 2016, American Chemical Society. All rights reserved. (b) Cycling stability of free-standing Li_2_S electrodes at 0.5C and a schematic illustration of their preparation using infiltration of active materials into porous carbonized biomass sheets. Reprinted with permission.^108^ Copyright © 2016 WILEY-VCH Verlag GmbH & Co. KGaA, Weinheim. All rights reserved.

Wu *et al.* applied four infiltration/vacuum-drying cycles to load Li_2_S into rGO, improving its distribution and promoting the formation of smaller Li_2_S nanoparticles, and then deposited a protective carbon layer by CVD to form a core–shell Li_2_S@C nanocomposite (Fig. 10a).^107^ The conductive graphene framework enhanced rate capability, while the vapor-deposited carbon shell together with an *in situ* formed passivation layer provided effective protection during cycling. As a result, the composite cathode retained approximately 97% of its initial capacity (∼1040 mA h g^−1^ (S) at 0.5C) after 700 cycles, demonstrating exceptional long-term stability.

They further extended this strategy to fabricate freestanding Li_2_S electrodes (Fig. 10b).^108^ Porous cellulose sheets were used as the scaffold and carbonized at 500 °C. Carbonization mitigated Li_2_S hydrolysis during processing and generated additional porosity, increasing surface area and facilitating Li_2_S loading and ion transport. The resulting cells exhibited minimal capacity decay at 0.5C.

A key limitation of this strategy is the very low solubility of Li_2_S in common solvents. For example, the solubility of Li_2_S in the most widely used solvent, ethanol, is around 25 mg mL^−1^ (∼0.5 M).^104–106,108–114^ This limited solubility constrains the amount of Li_2_S that can be infiltrated into the nanopores of porous carbon hosts, so a substantial fraction tends to precipitate outside the pore network. As a result, Li_2_S@C composites produced by this route often employ two-dimensional hosts, such as graphene-based frameworks, to facilitate uniform deposition and electrical contact.^104–108^

### 3.7 Precursor solution infiltration–decomposition method

**Fig. 11:**
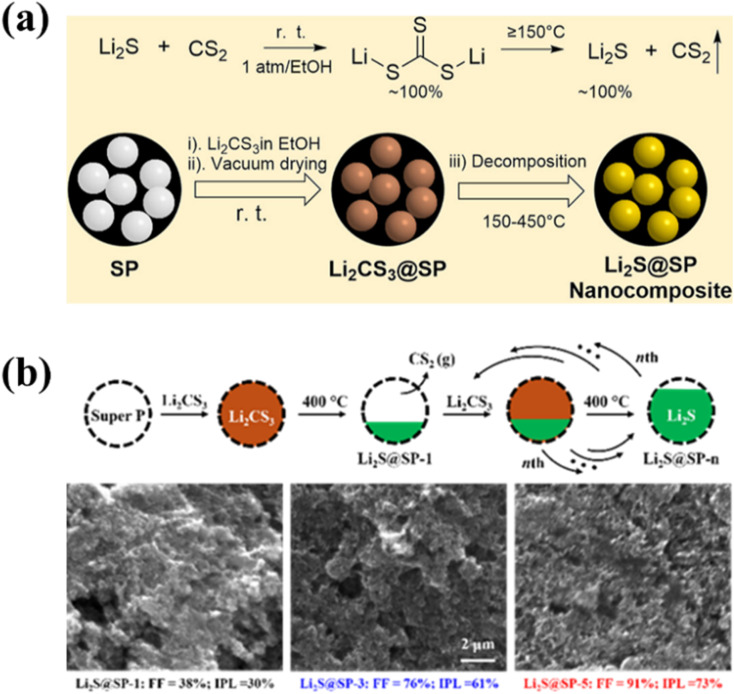
(a) Preparation of Li_2_S@SP nanocomposites through a precursor solution infiltration–decomposition method: (i) mixing SP with a Li_2_CS_3_ solution in anhydrous ethanol at room temperature, (ii) vacuum drying at room temperature, and (iii) thermal decomposition at 200–450 °C. Reprinted with permission.^65^ Copyright © 2025, American Chemical Society. All rights reserved. (b) Schematic illustration of the synthesis of Li_2_S@C nanocomposites with high in-pore Li_2_S loading *via* a multi-cycle Li_2_CS_3_ infiltration–decomposition strategy. This strategy achieves high in-pore Li_2_S loading and uniform distribution, leading to superior performance in lithium–sulfur batteries. Reprinted with permission.^115^ Copyright © 2026, American Chemical Society. All rights reserved.

To overcome the limited solubility of Li_2_S in conventional solvents, which restricts direct solution infiltration, we recently developed a precursor solution infiltration–decomposition method. As shown in Fig. 11a, Li_2_S is first converted to lithium trithiocarbonate (Li_2_CS_3_) *via* reaction with CS_2_ in ethanol at room temperature. The resulting Li_2_CS_3_ is highly soluble and largely amorphous, which greatly facilitates its infiltration into the mesoporous carbon host, Super P (SP).^65^ Subsequent thermal decomposition regenerates a Li_2_S@SP nanocomposite with Li_2_S confined within the mesoporous carbon framework. Thermal decomposition of Li_2_CS_3_ at 400 °C produced Li_2_S@SP-400 nanocomposites with ∼11 nm Li_2_S uniformly confined in Super P. The cathodes delivered a high discharge capacity of 821 mA h g^−1^ (Li_2_S) (∼1190 mA h g^−1^ (S)) with improved rate and cycling performance compared to commercial Li_2_S and melt-infiltrated S@SP, demonstrating the effectiveness and scalability of this nanoconfinement strategy. This simple, low-cost strategy is broadly applicable to diverse porous carbons, enabling uniformly dispersed Li_2_S@C nanocomposites with intimate interfacial contact, and offering a practical pathway toward Li_2_S-LSBs.

Nonetheless, thermal decomposition of Li_2_CS_3_ releases a substantial amount of CS_2_ gas (∼62 wt% of the precursor), which generates internal voids and limits in-pore Li_2_S formation. As a result, the in-pore filling is only ∼38%, with a significant fraction of Li_2_S deposited outside the pores.

To increase the in-pore loading, a multi-cycle infiltration strategy was developed (Fig. 11b).^115^ In this approach, Li_2_CS_3_ infiltration and decomposition are repeated for multiple cycles, allowing the newly created void space to be refilled in subsequent steps. This sequential “infiltrate–decompose–refill” process increased the pore-filling factor to 91% after five cycles. SEM and elemental mapping of the first-charged Li_2_S@SP-5 cathode confirm a homogeneous sulfur distribution within the carbon matrix, indicating uniform Li_2_S confinement and effective nanoscale dispersion throughout the SP framework. The resulting Li_2_S@SP-5 nanocomposite exhibited reduced activation overpotential, improved charge-transfer kinetics, and enhanced cycling stability, demonstrating the multi-cycle method as an effective and scalable route to high-loading, high-performance Li_2_S cathodes. Li_2_S@SP-5 retained 376 mA h g^−1^ after 500 cycles, compared with an initial capacity of 598 mA h g^−1^ at 1.0C, representing a significant improvement over the cell prepared with a single infiltration step.

As discussed above, considerable efforts have been devoted to developing Li_2_S@C nanocomposites through various synthesis strategies, including ball milling, carbothermal reduction, lithiation of sulfur/carbon composites, solution infiltration, and other advanced approaches. These strategies differ significantly in terms of Li_2_S loading, particle size control, structural design, synthesis complexity, and electrochemical performance. To provide a clearer overview and facilitate comparison, Table 3 summarizes the key synthesis strategies and corresponding electrochemical performances discussed in this review.

**Table 3:** Representative examples of Li_2_S@C nanocomposites prepared by different synthesis strategies

Strategy	Cathode	Li_2_S content (wt%)	Loading (mg cm^−2^)	Activation voltage (V)	Initial capacity (mA h g^−1^)	Capacity after cycling (mA h g^−1^)	Ref.
Ball milling	Li_2_S-C	67.5	0.54	4.0	1144@0.02C	411@0.1C@50 cycles	73
Ball milling	Li_2_S/CB@NC	72	∼1	4.0	1029@0.2C	652@0.2C@100 cycles	74
Ball milling	Li_2_S/C	74	3–3.5	4.0	971@0.1C	570@0.1C@200 cycles	75
Ball milling	Li_2_S/C/SnS_2_	75	∼1	∼3.5	712@0.1C	391@0.1C@100 cycles	76
Ball milling	Li_2_S-C-PVP	60	∼1.5	∼4.2	∼500@0.1C	∼460@0.1C@50 cycles	116
Carbothermal reduction	Li_2_S-C	72	2	3.8	600@0.2C	400@0.2C@200 cycles	78
Carbothermal reduction	Li_2_S@C-CNT	N/A	1.86	3.2	805@0.1C	595@0.2C@150 cycles	79
			3.7 (high loading)				
Carbothermal reduction	Li_2_S@C	62	0.54	3.0	330@0.5C	280@0.5C@40 cycles	81
Carbothermal reduction	Li_2_S/KB	68–78	3.5–4.0	3.4	938@0.1C	∼650@140cycles	82
Carbothermal reduction	Li_2_S	60	1.0	4.0	643@0.05C	459@0.05C@100 cycles	84
Carbothermal reduction-derived methods	Li_2_S@NCNF	50.6	3.0	3.5	720@0.2C	597.6@0.2C@50 cycles	85
Carbothermal reduction-derived methods	Li_2_S@Li_2_S_2_	60	∼1	4.0	∼750@0.05C	>400@0.5C@200 cycles	89
Carbothermal reduction-derived methods	TG-Li_2_S	53	1.3	∼3.4	1119@0.1C	791@0.1C@100 cycles	117
Carbothermal reduction-derived methods	Li_2_S@C-LPS-AB	38	1.75	2.4	680@2.0 mA cm^−2^	632@2.0 mA cm^−2^@700 cycles	118
Lithiation of S@C nanocomposites	PPy/Li_2_S/KB	N/A	N/A	4.0	∼850@0.2C	∼700@0.2C@80 cycles	90
Lithiation of S@C nanocomposites	Li_2_S/CMK-3	N/A	N/A	2.8	573@C/8	∼310@C/8@20 cycles	91
Lithiation of S@C nanocomposites	Li_2_S/KB/CNT	∼83.8	N/A	4.0	∼977@0.1C	414@0.1C@100 cycles	92
Lithiation of S@C nanocomposites	Li_2_S/GO@C	60	0.7–0.9	4.0	964@0.2C	∼700@0.2C@50 cycles	93
Lithiation of S@C nanocomposites	Li_2_S@C	60	1.0–1.5	4.0	972@0.2C	737@0.2C@100 cycles	94
Lithiation of S@C nanocomposites	Nano-Li_2_S/GA	69	3.66	3.6	838.5@0.1C	462.8@0.1C@100 cycles	95
Lithiation of S@C nanocomposites	Li_2_S (ALD)	67	N/A	3.0	∼860@55 mA g^−1^	∼800@55 mA g^−1^@36 cycles	96
Sulfuration of lithium compounds	Li_2_S-C	71.6	∼1.4	3.5	∼650–770@117 mA g^−1^	540@117 mA g^−1^@200 cycles	97
Sulfuration of lithium compounds	Li_2_S/C	∼56	∼2.68	4.0	∼770@0.2C	>410@0.2C@100 cycles	98
Sulfuration of lithium compounds	Li_2_S@graphene	∼88	10	3.5	1160@0.05C	>600@0.1C@200 cycles	99
Sulfuration of lithium compounds	Li_2_S@PC-CNT	68.2	1.34	3.8	1017@0.1 A g^−1^	502@0.5 A g^−1^@300 cycles	101
Sulfuration of lithium compounds	Li_2_S@C	92	N/A	4.0	1163@0.05C	954@0.1C@100 cycles	102
Sulfuration of lithium compounds	Li_2_S-rGO	∼66	∼0.96	3.5	982@0.1C	315@0.1C@100 cycles	119
Sulfuration of lithium compounds	Li_2_S-KB	71	3–3.2	3.4	∼868@0.05C	∼566@0.1C@100 cycles	120
Sulfuration of lithium compounds	HNG-Li_2_S	60	∼1.2	3.8	1067@0.05C	596@0.2C@500 cycles	121
Sulfuration of lithium compounds	Carbon-coated Li_2_S	51.3	∼1	∼3.2	∼700@0.1C	∼800@0.1C@15 cycles	122
Solution infiltration of Li_2_S	Graphene-Li_2_S	82–94	∼1	4.0	∼759@0.05C	∼698@0.1C@100 cycles	104
Solution infiltration of Li_2_S	Li_2_S/rGO	50–60	0.8–1.5	3.6	1119@0.1C	692@0.5C@145 cycles	105
Solution infiltration of Li_2_S	CNT/graphene-Li_2_S	81.4	∼4.0	3.6	1052.1@0.2C	958.3@0.2C@300 cycles	106
Solution infiltration of Li_2_S	Graphene-Li_2_S@C	55	1.3	2.8	742@0.2C	∼719@0.5C@700 cycle	107
Solution infiltration of Li_2_S	Li_2_S/C	∼50	∼1.3	2.8	∼870@0.5C	∼772@0.5C@200 cycles	108
Solution infiltration of Li_2_S	C-Li_2_S	∼73	∼1.4	3.8	947@0.2C	828@0.2C@100 cycles	109
Solution infiltration of Li_2_S	Li_2_S/N-doped graphene	50–55	∼2	4.0	801@0.3C	635@0.3C@100 cycles	110
Solution infiltration of Li_2_S	Li_2_S/graphene	∼50	1	3.6	894.7@	784.7@0.2C@300 cycles	111
Solution infiltration of Li_2_S	Li_2_S@Ni-P-S@G cage	60.6	5.2	4.0	980@0.1C	543@4C@100 cycles	112
Solution infiltration of Li_2_S	C-Li_2_S@C	∼60	N/A	2.8	∼850@0.2C	∼850@0.2C@300 cycles	113
Solution infiltration of Li_2_S	Li_2_S@C-Co-N	∼52	2	3.6	1137.1@0.2C	929.6@0.2C@300 cycles	114
Solution infiltration of Li_2_S	C-Li_2_S	90	1.35–1.62	3.8	826.1@0.1C	∼677@1C@500 cycles	123
Solution infiltration of Li_2_S	Nano-Li_2_S@C	72.3	2.8	2.8	1083.5@0.2C	766.4@0.2C@200 cycles	124
Solution infiltration of Li_2_S	3D-rGO-Li_2_S@C	75	2.5–3.5	3.8	856@0.1C	563.2@0.1C@100 cycles	125
Solution infiltration of Li_2_S	CF-CB-Li_2_S@C	N/A	7	3.8	943.7@0.1C	567.5@1C@200cycles	126
Precursor solution infiltration-decomposition method	Li_2_S@SP-400	60	1.0–1.2	4.0	821@0.1C	411@0.1C@100 cycles	65
Precursor solution infiltration-decomposition method	Li_2_S@SP-5	70	1.0–1.2	4.0	807@0.1C	402.5@0.1C@200 cycles	115

## 4 Li_2_S@C cathodes for all-solid-state batteries

Li_2_S cathodes are particularly attractive for all-solid-state Li–S batteries (ASSLSBs) because Li_2_S is already in the fully lithiated state and can therefore be paired with lithium-free anodes, enabling safer and more practical full-cell configurations. In addition, replacing flammable liquid electrolytes with solid electrolytes can effectively suppress polysulfide dissolution and improve battery safety.^35,127–129^ Therefore, integrating Li_2_S cathodes with solid-state electrolytes represents a promising route toward high-energy-density and safer sulfur-based batteries.

However, Li_2_S activation, which is a major challenge even in conventional liquid-electrolyte systems, has become even more difficult in solid-state batteries. In liquid-electrolyte cells, electrolyte penetration and soluble polysulfide intermediates can partially assist Li_2_S oxidation by providing continuous ion transport and additional reaction pathways. These electrolyte-mediated processes can, to some extent, compensate for the sluggish reaction kinetics of Li_2_S.^53^ In contrast, all-solid-state systems rely primarily on direct solid–solid interfacial reactions, where the absence of liquid-phase transport and soluble redox intermediates significantly increases the kinetic barrier for Li_2_S activation. As a result, the already challenging oxidation process of Li_2_S in liquid systems becomes even more demanding under solid-state conditions.

**Fig. 12:**
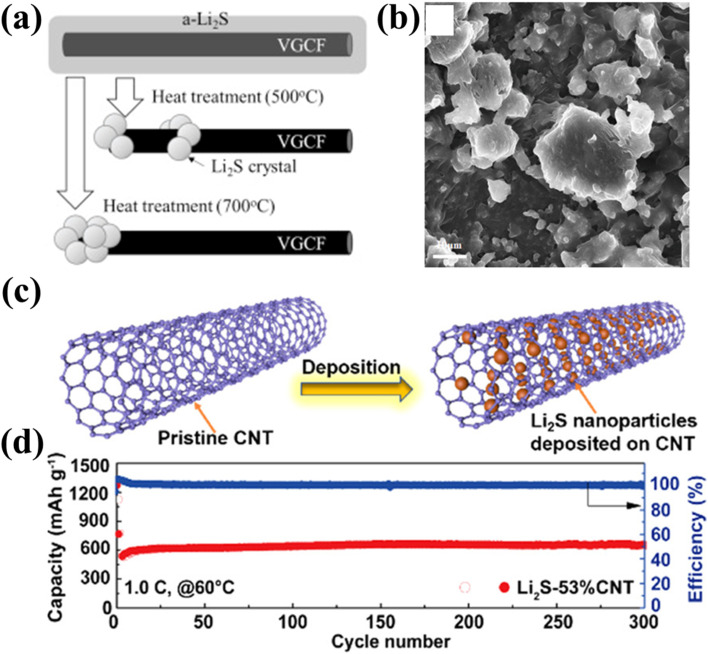
(a) Schematic of the nanocomposite evolution *versus* temperature. Reprinted with permission.^130^ Copyright© 2017 Elsevier B.V. All rights reserved. (b) SEM image of the as obtained Li_2_S@NC composite. Reprinted with permission.^131^ Copyright© 2020 Elsevier B.V. All rights reserved. (c) Schematic illustration of the preparation process for the Li_2_S-CNT cathode. (d) Cycling stability of the Li_2_S-53%CNT cathode at 1.0C and 60 °C. Reprinted with permission.^132^ Copyright © 2022, American Chemical Society. All rights reserved.

Eom *et al.* developed a Li_2_S–VGCF nanocomposite using a solution-assisted synthesis route followed by thermal treatment, in which nanosized Li_2_S particles were uniformly dispersed within a vapor-grown carbon fiber network (Fig. 12a).^130^ The cathode composite, prepared by mixing Li_2_S–VGCF with a Li_2_S–P_2_S_5_ solid electrolyte, was used to assemble ASSLSBs with Li_2_S–P_2_S_5_ as the solid electrolyte and a Li–In alloy as the anode. Compared with cells using oxide-based cathodes, these cells showed reduced interfacial resistance and avoided irreversible capacity loss. The multidimensional conductive framework improved electron transport and maintained stable conductive pathways during cycling, leading to capacities approaching 600 mA h g^−1^ with stable cycling up to 20 cycles. Wang *et al.* used an N-doped carbon-coated Li_2_S nanocomposite, Li_2_S@NC, to prepare a Li_2_S cathode for ASSLSBs using Li_7_P_3_S_11_ as the solid electrolyte and Li–In as the anode. In Li_2_S@NC, a thin nitrogen-doped carbon shell enhanced electrical conductivity and facilitated Li-ion transport (Fig. 12b). The resulting batteries achieved high Li_2_S utilization (∼91%) even at practical loadings exceeding 8 mg cm^−2^ and a capacity retention of 80% after 100 cycles.^131^ Jiang *et al.* synthesized ultrasmall Li_2_S nanoparticles (∼15 nm) anchored on CNTs using a liquid-phase method by mixing Li_2_S and CNTs in ethanol.^132^ The nanoscale Li_2_S and one-dimensional CNT conductive framework effectively improved reaction kinetics and reduced transport resistance. ASSLSBs with a Li/75%Li_2_S-24%P_2_S_5_-1%P_2_O_5_/Li_10_GeP_2_S_12_/Li_2_S-53%CNT architecture achieved a high initial capacity of 1160 mA h g^−1^ at 0.1C and retained a capacity of 651.4 mA h g^−1^ at 1C after 300 cycles at 60 °C.

Catalytic strategies have been explored to overcome the sluggish solid–solid conversion kinetics of Li_2_S cathodes in ASSLSBs. By introducing catalytic species into Li_2_S cathodes, the activation barrier of Li_2_S can be reduced and the conversion between Li_2_S and sulfur can be accelerated, thereby improving active-material utilization and long-term cycling stability. Liu *et al.*^133^ developed an ASSLSB using a solid polymer electrolyte through *in situ* polymerization of DOL and a Li_2_S@Co-C@MHF cathode, which was prepared by first thermally infiltrating elemental sulfur into Co nanoparticle-decorated carbon nanocages encapsulated within MXene hollow fibers (Co-C@MHF), followed by converting sulfur into Li_2_S using lithium naphthalide. The metallic Co in the cathode served as catalytic sites to facilitate Li_2_S activation and sulfur conversion while MXenes improved charge transport and confined reaction intermediates. The catalytic effect significantly reduced the Li_2_S activation barrier to ∼2.32 V and enabled stable cycling over 500 cycles with high-capacity retention. Similarly, Hao *et al.*^134^ prepared nanosized Li_2_S embedded within an amorphous LiFeS_2_ matrix (Li_2_S@LiFeS_2_) through an *in situ* solid-state reaction between Li_2_S and FeCl_3_ using ball milling and mixed it with Li_6_PS_5_Cl and carbon black to prepare a cathode composite. The amorphous LiFeS_2_ simultaneously functioned as a catalytic phase and a mixed ionic/electronic conductor, while the solid electrolyte Li_6_PS_5_Cl improved the lithium-ion conductivity within the cathode. The battery using Li_6_PS_5_Cl as the solid electrolyte showed substantially improved transport properties and nearly 99% capacity retention after 300 cycles. However, the discharge voltages are between 1.6 and 0.8 V, significantly lower than that of the liquid electrolyte LSBs (between 2.4 and 1.7 V), reducing cathode energy density.

Redox mediators have also been used to improve the kinetics of ASSLSBs. Yu *et al.*^135^ introduced In_2_S_3_ as a mediator into Li_2_S cathodes, forming Li_2_S–Li_*x*_In_2_S_3_ composites during cycling. Li_2_S–Li_*x*_In_2_S_3_ was prepared by ball milling Li_2_S with In_2_S_3_, then with vapor-grown carbon fiber (VGCF), and finally with a solid electrolyte Li_7_P_3_S_11_. ASSLSBs with this cathode were assembled with a Li_7_P_3_S_11_ solid electrolyte and a Li/In anode. The battery performance indicated that Li_*x*_In_2_S_3_ simultaneously functioned as a redox mediator and a charge-carrier mediator for Li^+^ and electrons, improving transport kinetics and actively regulating electrode volume variation.

Compared with catalyst-assisted systems that mainly lower reaction barriers at interfaces, mediator-assisted strategies actively participate in electrochemical processes and create alternative reaction pathways, simultaneously improving reaction kinetics and transport characteristics. However, ensuring long-term mediator stability while minimizing inactive mass remains an important consideration for practical applications.

It should be noted that most Li_2_S cathodes reported for ASSLSBs are based on Li_2_S/C composites, that is, physical mixtures of Li_2_S and conductive carbon, rather than true Li_2_S@C nanocomposites. This is presumably due to the synthetic challenges associated with preparing Li_2_S@C nanocomposites. Although catalyst- and mediator-assisted activation approaches can effectively improve Li_2_S oxidation, they may not simultaneously address the intrinsic limitations of Li_2_S, including poor electronic conductivity and severe electrochemical polarization. Therefore, integrating catalyst- or mediator-assisted activation strategies with Li_2_S@C nanocomposites may provide additional advantages for all-solid-state systems.

## 5 Conclusions and future developments

LSBs are widely considered as promising next-generation energy-storage systems because of their high theoretical energy density, low cost, material abundance, and environmental compatibility. However, intrinsic limitations of sulfur cathodes, including extremely low electronic/ionic conductivity, large volume change during cycling, and the polysulfide shuttle, have long constrained commercially viable performance. Over the past decade, substantial progress has been made to address these challenges, and near-commercial metrics have been demonstrated in certain reports. Most of these advances, however, have been achieved in S-LSBs, whose practical deployment remains largely hindered by the use of lithium-metal anodes.

In this context, Li_2_S-LSBs have drawn increasing attention because they can, in principle, eliminate lithium-metal anodes. Li_2_S cathodes may also help mitigate cathode volume change and, to some extent, reduce polysulfide dissolution, offering potential benefits for cycle life. Despite sharing the same overall sulfur redox chemistry, Li_2_S-LSBs face distinct challenges, notably the moisture sensitivity of Li_2_S (complicating electrode fabrication and handling) and the well-known first-charge activation overpotential, which often leads to lower accessible capacity and poorer cycling stability. As a result, Li_2_S-LSBs still lag well behind S-LSBs in overall maturity. Meanwhile, recent developments in all-solid-state batteries have also attracted growing interest toward Li_2_S cathodes because solid electrolytes can potentially suppress polysulfide dissolution and improve cell safety.

A primary origin of the high overpotential and suboptimal performance is the difficulty of preparing well-defined Li_2_S@C nanocomposites in which Li_2_S is uniformly embedded within nanoscale porous carbon hosts. Unlike S@C nanocomposites, which can be conveniently produced *via* melt infiltration, Li_2_S has a high melting point and is challenging to thermally infiltrate into porous frameworks. As reviewed here, a range of physical and chemical routes has been explored for Li_2_S@C synthesis, including ball milling, carbothermal reduction and derived routes, lithiation of preformed S@C nanocomposites, sulfuration of lithium-containing precursors, solution-based infiltration of Li_2_S, and the precursor solution infiltration–decomposition method.

Among these routes, ball milling is simple but energy intensive, typically cannot produce Li_2_S particles below ∼100 nm and is ineffective for deeply embedding Li_2_S into mesoporous hosts. Solution infiltration is straightforward and can be effective for 2D hosts (*e.g.*, graphene), but the low solubility of Li_2_S limits infiltration into nanoporous carbons. Carbothermal approaches using inexpensive sulfur-containing compounds can form Li_2_S@C *in situ* and offer scalability potential, although high processing temperature/energy consumption and limited architectural control remain concerns. Sulfuration of lithium compounds using H_2_S, CS_2_, or sulfur is another promising scalable strategy, but constructing suitable precursor@C architectures and achieving complete conversion require further efforts. Direct lithiation of preformed S@C nanocomposites can yield well-defined nanostructures but commonly relies on hazardous and costly lithiating reagents (*e.g.*, butyllithium), compromising practicality. Notably, the precursor solution infiltration–decomposition method using *in situ* generated Li_2_CS_3_ from Li_2_S and CS_2_ appears low-cost, operationally simple, and potentially scalable. It can produce Li_2_S particles with sizes governed by the carbon host pore structure (*e.g.*, ∼30 nm with Super P) and with uniform distribution. Li_2_S-LSBs prepared *via* this route have delivered performance comparable to, or better than, S-LSBs using melt infiltrated S@C, demonstrating the promise of this method for advancing Li_2_S-LSBs toward practical implementation.

Beyond conventional liquid-electrolyte systems, recent studies have extended Li_2_S cathodes into all-solid-state battery configurations. Unlike liquid-electrolyte Li_2_S-LSBs, where suppressing polysulfide dissolution remains a major focus, the development of Li_2_S cathodes for ASSLSBs faces additional challenges associated with sluggish solid-state reaction kinetics. Existing studies have mainly focused on confining nanosized Li_2_S particles on CNTs and carbon fibers and introducing redox catalysts or mediators to improve ionic/electronic transport, reaction pathways, and conversion kinetics. Nonetheless, the application of Li_2_S@C nanocomposites in ASSLSBs remains relatively limited. Rational design of Li_2_S@C architectures is expected to provide more effective strategies for improving sulfur utilization and electrochemical performance in all-solid-state systems.

Besides Li_2_S@C synthesis, additional bottlenecks must be addressed before Li_2_S-LSBs can be commercialized. Cycle stability remains generally inferior to that of S-LSBs, with the polysulfide shuttle still being a dominant degradation pathway. While numerous shuttle-mitigation strategies have been developed for S-LSBs, their implementation in Li_2_S cathodes is constrained by Li_2_S's moisture sensitivity and chemical reactivity. For example, aqueous-processable binders^136^ are incompatible with Li_2_S electrode fabrication, and some functional binders contain groups that readily react with Li_2_S.^25^ Even PVDF, the most widely used stable binder, has been reported to react with Li_2_S during slurry preparation, consuming active material and degrading performance.^46^ Consequently, only a limited set of binders has been successfully adopted in Li_2_S-LSBs,^45^ and many catalytic or polysulfide-trapping additives effective in S-LSBs cannot be directly incorporated due to undesirable side reactions.

Future progress in Li_2_S-LSBs will likely rely on: (i) optimizing existing Li_2_S@C synthesis routes and developing more practical, scalable methods; (ii) developing Li_2_S-compatible functional binders and additives that simultaneously immobilize polysulfides and accelerate redox kinetics; and (iii) advancing cell-level engineering, including reduced electrolyte-to-sulfur ratios and full-cell configurations with lithium-free anodes. In parallel, advances in solid electrolytes and Li_2_S@carbon nanocomposite design may further accelerate the development of all-solid-state Li_2_S-LSBs. Future studies should focus on improving solid-state Li_2_S reaction kinetics, stabilizing cathode/electrolyte interfaces, and constructing effective ionic/electronic transport networks. Given the currently limited number of reported Li_2_S@carbon nanocomposite systems in all-solid-state batteries, further development of nanoscale Li_2_S confinement strategies and optimized carbon architectures represents a promising direction for future research. These developments could ultimately provide a pathway toward high stability, improved safety, and high energy density by fundamentally suppressing polysulfide shuttle and enabling efficient solid-state sulfur redox reactions.

## Author contributions

Zhe Huang: conceptualization, data curation, formal analysis, investigation, validation, visualization, writing – original draft, writing – review and editing. Yixuan Zhao: data curation, formal analysis. Yonglin Wang: data curation, formal analysis. Yuning Li: conceptualization, formal analysis, funding acquisition, investigation, visualization, project administration, resources, supervision, writing – review and editing.

## Conflicts of interest

There are no conflicts to declare.

## Data Availability

No new data were generated or analyzed in this study. Data sharing is not applicable to this article.
